# Trend Analysis of Maternal Mortality in Kenya: Post-Devolution Empirical Results

**DOI:** 10.24248/eahrj.v7i2.728

**Published:** 2023-11-30

**Authors:** Oluwafunmilola Deborah Awe, Hillarry Kipruto, Olawale Awe, Queensley C. Chukwudum

**Affiliations:** a Department of Medical Statistics (UNITID), University of Nairobi, Kenya; b Senior Statistician, World Health Organization, Nairobi, Kenya; c Department of Mathematical Sciences, Anchor University Lagos; d Department of Insurance and Risk Management, University of Uyo, Akwa Ibom State, Nigeria

## Abstract

**Introduction:**

Kenya has taken significant steps to improve Reproductive Maternal Neonatal Child Adolescent Health (RMNCAH) services, with a vision to prevent preventable deaths of women and newborns. This study seeks to fill a crucial gap in understanding the dynamics of maternal mortality in Kenya, post-devolution

**Materials and Methods:**

The study spans all the 47 counties of the Republic of Kenya, using county as the unit of analysis. Maternal Mortality Ratio (MMR) data was extracted from the District Health Information Software (DHIS), which was created to evaluate the level of progress in coverage of RMHCAH service in Kenya. Changes in the MMR Indicator was modelled over time using Repeated measures one-way ANOVA in the 47 counties in Kenya.

**Results:**

A descriptive study uncovered an average reduction in the MMR, which decreased from approximately 170 to 130 per 100,000 live births between 2012 and 2018. There was a steady decrease in MMR in Garisa, Mombasa, Busia, Elgeyo, Samburu and Uasin. Tables and figures were used to visualise findings.

**Conclusions:**

Our findings revealed that although there has been continuous improvement of relative equity over the last quarter-century in all the 47 counties in Kenya, uneven coverage remains within each county. This lack of equity differs from one county to another. There was a significant difference within each year and among the years, and pairwise comparison revealed a significant difference in the MMR between 2012 and all the years except 2016 and 2017.

## INTRODUCTION

Maternal death, as defined by the World Health Organization (WHO), refers to the demise of a woman due to pregnancy-related causes within 6 weeks of pregnancy termination for any reason, including management-related.^1,2^ The Maternal Mortality Ratio (MMR) is a key indicator, representing the number of maternal deaths per 100,000 live births within a specific time frame.^3^ Kenya has taken significant steps to improve Reproductive Maternal Neonatal Child Adolescent Health (RMNCAH) services, with a vision to prevent preventable deaths of women and newborns.

In 2010, Kenya initiated a devolution of healthcare services, transferring essential responsibilities from the national to the county level to enhance access to care. While there has been a noteworthy reduction in MMR in Kenya due to government efforts, gaps and inequalities in RMNCAH service provision persist. This necessitates continuous monitoring of maternal health progress after the devolution of health services in Kenya.

Globally, more than 500,000 women die annually due to pregnancy and childbirth-related complications, with approximately 50–62% of these deaths occurring in Sub-Saharan Africa.^4-7^ These statistics highlight the severity of the issue, with 830 women dying daily from such complications.^8^ Direct obstetric causes, which are preventable, account for 73–80% of maternal deaths, with indirect causes contributing to the remaining 28%.^9^ Notably, Sub-Saharan Africa has seen a shift in the global burden of MMR from Asia due to various factors.^10^

Hemorrhage, hypertensive disorders, and sepsis are responsible for over 50% of maternal deaths worldwide.^11,12^ In Sub-Saharan Africa, 65% of maternal deaths from hemorrhage occur following delivery.^13,14^ Recent studies have revealed that maternal mortality has decreased globally since 2000, but Sub-Saharan Africa still bears a disproportionate burden (67%) with Chad (1140/100,000 live births), Sierra Leone (1120/100,000) and South Sudan (1150/100,000) being the top 3 countries with the highest maternal mortality deaths.^31^ Factors such as prenatal care coverage, skilled personnel-assisted births, access to improved water resources, higher adult literacy rates, and adequate income have been linked to reduced maternal mortality.^32^

While Kenya does not rank among the top 10 African countries with the highest maternal mortality rates, it continues to experience a high MMR, despite government commitments to address the issue. Between 1990 and 2015, Kenya witnessed a minimal decrease in MMR, from 687 to 510 maternal deaths per 100,000 live births.^15,31^ County-wise data is limited, but a 2014 survey estimated a lower MMR of 362 per 100,000 live births and a lifetime risk of 1 in 67.^15^

However, there is considerable county-level variation. For instance, Mandera County recorded an exceptionally high MMR in 2008–09 (3,795/100,000), exceeding that of wartime Sierra Leone (20,000/100,000).^15^ Various studies have examined factors related to maternal mortality in Kenya, with a focus on specific populations.^33,34^ Notably, very little county-level research has been conducted. A 2014 Kenyan Demographic and Health Survey provided county-level data and concluded that MMR declined insignificantly between 2008 and 2014. Variations in maternal health indicators exist across counties, emphasizing the need for localized strategies.^35^

Despite increased facility-based deliveries in Kenya, MMR and neonatal mortality rates have remained relatively unchanged.^36^ Access to proven maternal health services, including prenatal, delivery, and postnatal care, is essential to reduce these life-threatening complications.^8^

The United Nations' Millennium Declaration aimed to reduce MMR by 75% from 1990–2015, emphasizing the importance of universal access to reproductive health.^16^ To achieve this goal, adequate political participation, financial resources, and realistic strategies are essential.

Most studies in the literature have focused on MMR in the pre-devolution era, with limited examination of changes during the post-devolution of health services in Kenya. Notably, no research has explored the trend and spatiotemporal aspects of MMR at the county level post-devolution. To address this gap, this study examines the trend in MMR using repeated measure analysis for 47 counties post devolution (2012-2018). This micro-level investigation allows for county-to-county comparison, revealing variations in maternal health coverage and enabling better planning.

This study seeks to fill a crucial gap in understanding the dynamics of maternal mortality in Kenya, post-devolution. By examining trends and variations at the county level, it contributes valuable insights for more effective maternal health planning and policies. The results and discussions of this analysis will provide a foundation for targeted interventions to further reduce MMR in Kenya.

## MATERIALS AND METHODS

The study spans all the 47 counties of the Republic of Kenya, using county as the unit of analysis divided into 8 administrative provinces Central, Coast, Eastern, Nairobi, North Eastern, Nyanza, Rift Valley and Western Province.^23^ MMR data was extracted from the District Health Information Software (DHIS), which was created to evaluate the level of progress in coverage of RMHCAH service in Kenya.^24^ Changes in the MMR Indicator was modelled over time using Repeated measures one-way ANOVA in the 47 counties in Kenya. A spatio-temporal analysis was also done. Analysis was done using R software version 3.3.1, QGIS and SPSS version 13.

### Repeated Measure Designs

They are a type of General Linear model, an extension of the paired t-test useful when samples are matched based on important features. The matched groups have equal sample size and are exposed to a level of factor or group of factors. There is the within subjects and between-subjects factor which means the outcome variable is repeatedly measured for all members of the sample across a range of conditions and measurement of independent group members respectively. Thus a repeated measures ANOVA consists of these 2 factors explained above. The utility of this model is in its' excellent precision capability when comparing time points or treatments within some subjects, thus eliminating all sources of variation between subjects with just within-subjects variability making up the experimental error. Subjects becomes their own controls. The disadvantage lies in its' order effect and carryover effect. There is assumption of normality of response variable, homogenous variance known as sphericity (i.e. a significant value, for the purpose of this study level of significance =0.05). Violation of this assumption, might not exclude the model use but reduces the power of the test. Alternatively, multivariate analysis can be used such as Hotelling-Lawley trace, Wilks' lambda and Pillai-Bartlett trace.^25-29^ There is a similarity between the test statistic for repeated-measures ANOVA and that for independent-measures ANOVA. Repeated measure can be One-way ANOVA or two-ANOVA depending on the number of independent variables included in the study for the purpose of this study One-way ANOVA (one variable) was utilised.^27^

#### Within-subject model



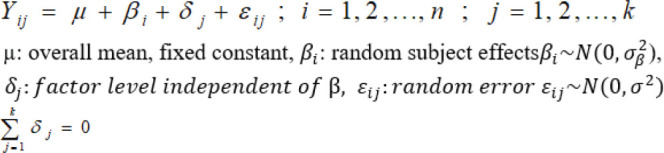



#### Between-subject model



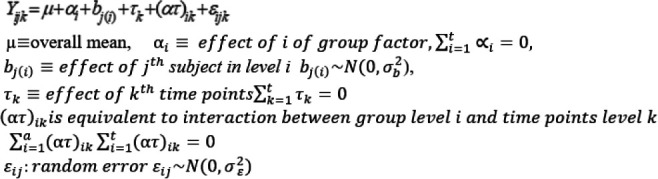



Mauchly's Test is used to check for Sphericity. If violations of sphericity do occur, corrections can be made such that a more valid critical *F*-value is gotten, thereby, reducing the Type I error rate. An estimation of the extent of sphericity violation is done and a correction factor is applied such as **Greenhouse-Geisser, Huynh-Feldt and lower-bound correction**. They correct this firstly by estimating the degree of sphericity using epsilon ε. When ε is equal to 1 then there is maximum sphericity. The more ε reduces (ε <1) the greater the extent of sphericity. The Huynd-Feldt and Greenhouse-Geisser methods estimate ε although using different methods. These 3 methods of correction adjust the degree of freedom by multiplying the degree of freedom using their individual ε estimates.



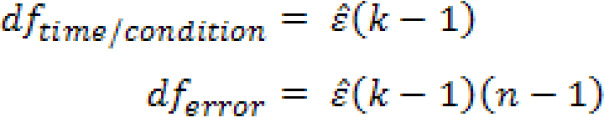



For further insights, Bonferroni t-tests were employed to conduct pairwise comparisons between the means of different years. This analysis aimed to identify the specific year or years that contributed to variations in factors influencing the maternal mortality rate, as demonstrated in Table 5. The Bonferroni test controls the overall error rate by establishing the error rate for each test in relation to the experiment-wise error rate, dividing it by the total number of tests.

### Spatio-temporal Analysis

This is a statistical technique that seeks to explain the when, where and sometimes why events occur. Several scientific researchers have utilized this method to give a better explanation of the geographical and time distribution of the event of interest.^29-30^ This study as utilized a very simple approach of mapping the changes in Maternal mortality over time across the 47 counties in Kenya.

## RESULTS

**FIGURE 1:**
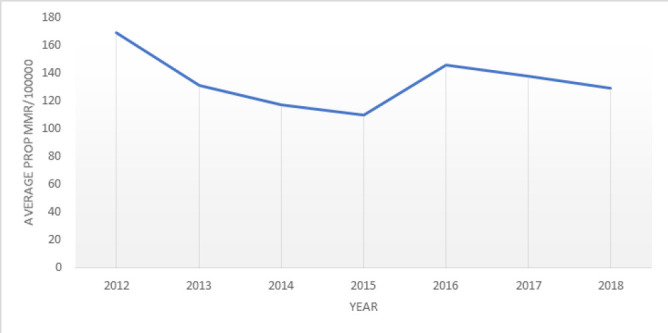
Trend of Total Proportion of MMR/100,000 Live Birth in Kenya (2012-2018)

**FIGURE 2:**
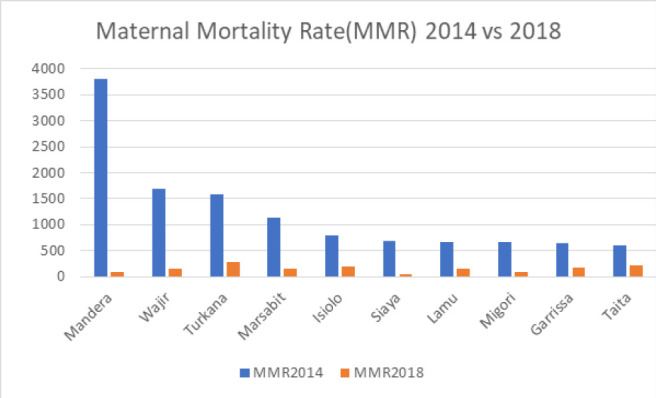
Comparison Between MMR in Ten Selected Kenyan Counties

**FIGURE 3:**
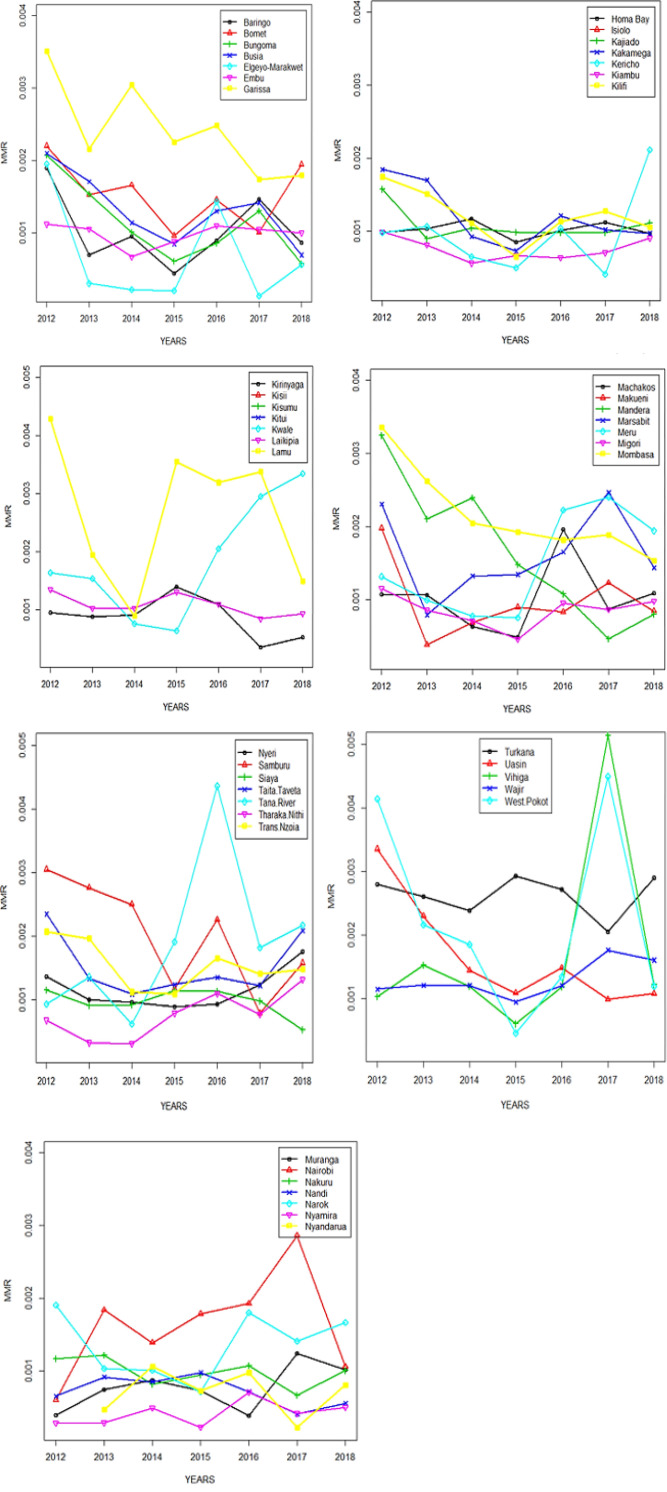
Line Plots for MMR Across Counties Grouped in an Alphabetical Order in Kenya from 2012–2018

**FIGURE 4:**
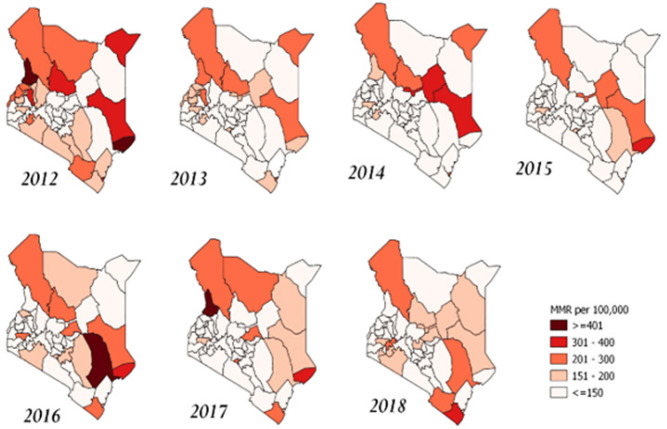
Spatial Maps of MMR/100 live Births in Kenya (2012-2018)

A descriptive study uncovered an average reduction in the Maternal Mortality Ratio (MMR), which decreased from approximately 170 to 130 per 100,000 live births between 2012 and 2018, as shown in Table 1, Figure 1. The examination of MMR trends in Kenya over this period revealed a consistent decline from 2012 to 2015, followed by a notable increase from 2015 to 2016, and subsequent gradual decrease, as depicted in Figure 2. There was a steady decrease in MMR in Garisa, Mombasa, Busia, Elgeyo, Samburu and Uasin. In 2016, there was a remarkable increase compared to the ratio they had from 2012–2015 in Siaya and Tana River. In 2017, there was a sharp increase in MMR in Vihiga, West-Pokot and Nairobi possibly contributed by the crisis during the election period and hospital strikes. There was a consistently low MMR in County Embu, Laikipia, and Migori with about 100 deaths/100,000 with Kiambu, Nyamira having a lower MMR below 100/100000 from 2012–2018 (Figure 3 and Figure 4).

**TABLE 1:** Proportion of MMR/100,000 Live Birth in Kenya

Year	MMR/100000
2012	169.21 (99.940)
2013	131.490 (63.040)
2014	116.970 (65.690)
2015	109.650 (70.480)
2016	145.620 (72.000)
2017	138.020 (102.980)
2018	129.230 (61.14)

To conduct a repeated measures one-way ANOVA, Mauchly's test of sphericity was performed after ensuring normality checks. The results of the test were significant, as displayed in Table 2. Consequently, a Greenhouse-Geisser correction was applied and also found to be significant, as presented in Table 3.

**TABLE 2:** Mauchly's Test of Sphericity

Within Subjects Effect	Mauchly W	Approx. Chi-Square	Df	Sig	Greenhouse-Geisser	Epsilon Huynh-Fiedt	Lowerbound
Years	0.127	90.207	20	**0.000**	0.648	0.715	0.167

**TABLE 3:** Significant Tests of Within-Subjects Effects for MMR in Kenya

Source		Type III Sum of Squares	df	Mean Square	F	Sig.
Years	Sphericity Assumed	108231.830	6	18038.638	4.790	.000
	Greenhouse-Geisser	108231.830	3.886	27850.768	4.790	.001
	Huynh-Feldt	108231.830	4.290	25231.680	4.790	.001
	Lower-bound	108231.830	1.000	108231.830	4.790	.034
Error (Years)	Sphericity Assumed	1039421.067	276	3766.018		
	Greenhouse-Geisser	1039421.067	178.762	5814.547		
	Huynh-Feldt	1039421.067	197.318	5267.746		
	Lower-bound	1039421.067	46.000	22596.110		

A linear trend analysis was conducted, and the results were statistically significant. This significance indicates that the observed differences across the years were not due to random chance, as detailed in Table 4.

**TABLE 4:** Trend Analysis for MMR in Kenya

Source	Years	Type III Sum of Squares	df	Mean Square	F
Years	Linear	10281.072	1	10281.072	1.572
Error (Years)	Linear	300877.431	46	6540.814	

The Bonferroni test revealed a significant difference between the year 2012 and all other years except for 2016 and 2017, with a significance level set at 0.05 (Table 5). In summary, urban counties such as Nairobi, Nakuru, Mombasa had steady decrease in Maternal mortality while rural counties such as Samburu, Tana River, Lamu had worsening MMR expectedly.

**TABLE 5:** First Pairwise Comparison Maternal Mortality Ratio in Kenya (Significant Result)

Year	(J) Years	Mean Difference	Std. Error	P-Value
2012	2013	37.726	10.660	.001
	2014	52.253	12.821	.000
	2015	59.568	14.215	.000
	2016	23.604	14.442	.109
	2017	31.200	16.603	.067
	2018	39.987	15.532	.013

In summary, urban counties such as Nairobi, Nakuru, Mombasa had steady decrease in Maternal mortality while rural counties such as Samburu, Tana River, Lamu had worsening MMR expectedly.

## DISCUSSION

The objective of this study was to address a notable knowledge gap concerning maternal mortality dynamics in Kenya following the process of devolution.^8^ The findings shed light on a critical aspect of maternal health, the MMR per 100,000 live births, over a six-year period spanning from 2012 to 2018.

The temporal trend analysis, conducted at the county level, revealed intriguing insights into the state of maternal health in Kenya. In 2012, certain counties, specifically Lamu, Garissa, Mombasa, and Mandera, exhibited alarmingly high MMR figures, with rates standing at 429, 351, 335, and 325 per 100,000 live births, respectively. These elevated MMRs can be attributed to the challenging living conditions and the nomadic lifestyle characteristic of residents in these areas. This highlights the importance of tailoring healthcare strategies to address the unique needs of these communities.

One notable positive aspect we observed is the overall improvement in MMR in Kenya, with a notable reduction to 110 per 100,000 live births in our 2018 data. This suggests that there has been progress in reducing maternal mortality following the devolution process. However, it is essential to acknowledge that disparities persist, as indicated by inequality in MMR reduction. ^8,17^ Counties like West Pokot, Vihiga, Turkana, and Kwale still reported alarmingly high MMR figures, with rates as high as 500 per 100,000 live births in 2018. Factors contributing to these disparities include lifestyle differences and the geographical location of inhabitants, which can impede accessibility to essential healthcare services.

Moreover, our findings underscore the urgent need for Kenya to intensify its efforts in the healthcare sector to meet global targets, such as those set in the Sustainable Development Goals (SDGs) by 2030.^15^ To achieve this Specific development goals specifically, SDG 3.1 (i.e. to reduce maternal mortality to less than 70 per 100,000 live births), it is imperative that Kenya's healthcare system provides more resources to improve access to quality healthcare. Also, the healthcare stakeholders must join hands to enact laws that will provide strategic interventions to ensure that maternal health disparities are addressed comprehensively. Our study calls for a renewed commitment to this cause to ultimately enhance the well-being of Kenyan mothers and align the nation with global health objectives.

### Limitations

Some limitations were observed in this study. One, there is limited control over the quality of secondary data. However, a follow up qualitative study was done after this study, with results that corroborated these findings. It will be published separately because of the extensive results. Secondly, missing data and extreme values are difficult to track as it is in the past. Finally, more advanced analysis could be done such as longitudinal analysis, fixed effect models etc. to know the difference in the counties more objectively.

## CONCLUSION

This article aimed to analyse the progress in Maternal Heath and Mortality in Kenya during the Post-devolution era (2012-2018). Our findings revealed that although there has been continuous improvement of relative equity over the last quarter-century in all the 47 counties in Kenya, uneven coverage remains within each county. This lack of equity differs from one county to another. There was a significant difference within each year and among the years, and pairwise comparison revealed a significant difference in the MMR between 2012 and all the years except 2016 and 2017. Thus, there appears to be some progress in MMR in Kenya. Also, there was a significant association between higher equity of service coverage, higher education, and the government's dedication to Reproductive Health and Maternal Health in Kenya. These results provide essential information about maternal and early neonatal mortality in Kenya, and they are useful for donors and policymakers in Kenya. These results provide important information about maternal mortality in Kenya and they are useful for donors and policy makers in Kenya.

In summary, although devolution appeared to have reduced maternal mortality rates to certain extent in Kenya, there is still gross inequality across counties because some counties are more badly affected than others. Therefore, policy makers in the affected counties should borrow ideas from successful counties like Nairobi.

## RECOMMENDATIONS

Policy makers should intensify policies that would prevent pregnancy-related mortality in affected counties by ensuring that all women have access to proven lifesaving maternal health services and prompt management of complications related to pregnancy and childbirth in all Kenyan Counties. Further studies will be carried out in future such as mixed methods, spatial and longitudinal studies to assess these differences across counties and their possible reasons. Additionally, it is essential to factor in specific causes of maternal deaths in future research, to emphasize particular areas that healthcare providers and policymakers should prioritize.
